# Plasma lipidomic profiling of thiopurine-induced leukopenia after NUDT15 genotype-guided dosing in Chinese IBD patients

**DOI:** 10.3389/fnut.2023.1138506

**Published:** 2023-06-27

**Authors:** Pan Li, Kang Chao, Zhanhua Hu, Lulu Qin, Ting Yang, Jing Mao, Xia Zhu, Pinjin Hu, Xueding Wang, Xiang Gao, Min Huang

**Affiliations:** ^1^Guangdong Provincial Key Laboratory of New Drug Design and Evaluation, School of Pharmaceutical Sciences, Sun Yat-sen University, Guangzhou, China; ^2^Department of Gastroenterology, The Sixth Affiliated Hospital, Sun Yat-sen University, Guangzhou, China; ^3^Guangdong Institute of Gastroenterology, Guangdong Provincial Key Laboratory of Colorectal and Pelvic Floor Diseases, Supported by National Key Clinical Discipline, Guangzhou, China; ^4^School of Pharmaceutical Sciences, Guangdong Pharmaceutical University, Guangzhou, China

**Keywords:** thiopurines, lipidomic, inflammatory bowel disease, biomarkers, machine learning

## Abstract

**Introduction:**

Thiopurines, azathiopurine (AZA) and mercaptopurine (6-MP) have been regularly used in the treatment of inflammatory bowel disease (IBD). Despite optimized dosage adjustment based on the NUDT15 genotypes, some patients still discontinue or change treatment regimens due to thiopurine-induced leukopenia.

**Methods:**

We proposed a prospective observational study of lipidomics to reveal the lipids perturbations associated with thiopurine-induced leukopenia. One hundred and twenty-seven IBD participants treated with thiopurine were enrolled, twenty-seven of which have developed thiopurine-induced leucopenia. Plasma lipid profiles were measured using Ultra-High-Performance Liquid Chromatography-Tandem Q-Exactive. Lipidomic alterations were validated with an independent validation cohort (leukopenia *n* = 26, non-leukopenia *n* = 74).

**Results:**

Using univariate and multivariate analysis, there were 16 lipid species from four lipid classes, triglyceride (*n* = 11), sphingomyelin (*n* = 1), phosphatidylcholine (*n* = 1) and lactosylceramide (*n* = 3) identified. Based on machine learning feature reduction and variable screening strategies, the random forest algorithm established by six lipids showed an excellent performance to distinguish the leukopenia group from the normal group, with a model accuracy of 95.28% (discovery cohort), 79.00% (validation cohort) and an area under the receiver operating characteristic (ROC) curve (ROC-AUC) of 0.9989 (discovery cohort), 0.8098 (validation cohort).

**Discussion:**

Our novel findings suggested that lipidomic provided unique insights into formulating individualized medication strategies for thiopurines in IBD patients.

## 1. Introduction

Inflammatory bowel disease (IBD), including Crohn's disease (CD) and ulcerative colitis (UC), is characterized by chronic and relapsing intestinal inflammation (1). The incidence of IBD is accelerating due to more Westernized societies in China (1, 2). IBD is affecting millions of people worldwide, and IBD-related healthcare costs have increased significantly (3, 4).

Thiopurines, AZA and 6-MP, have been conventionally used to maintain remission as steroid-sparing agents in IBD patients (5–8). However, 10–30% of patients discontinue therapy due to adverse events (8–10). The most frequent and severe adverse event is leukopenia (thiopurine-induced leukopenia, TIL), especially in Asian populations (11, 12). The Clinical Pharmacogenetics Implementation Consortium has developed guidelines for guiding thiopurine drug dosing based on *TPMT* and *NUDT15* genotypes (13). Thiopurine methyltransferase (TPMT), as a biomarker of thiopurine drug toxicity, effectively solves the problem of leukopenia in European and American populations. However, the mutation rate of *TPMT* in Asian populations is extremely low (~1%) and cannot effectively guide the dosing regimen for the Asian population (14, 15). The nucleic acid oxidation inhibitor gene (*NUDT15*) in Asian populations plays a vital role in TIL (16–19). The loss-of-function allele in the *NUDT15* gene, which is mutated at a high frequency in Asians, decreases the degradation of active thiopurine nucleotide metabolites and leads to leukopenia (13). Thiopurines are not recommended as a treatment option in patients with *NUDT15 TT* because leukopenia is certain to occur in this patient (12). Although our previous prospective, randomized, controlled trial confirmed that dose optimization according to *NUDT15 C415T* could reduce the incidence of TIL, without significantly affecting efficacy (5), there were still 23.7% of patients developed thiopurine-induced leucopenia (5). It is noted that drug metabolism including the active product 6TGN and metabolic enzyme genotype *TPMT/NUDT15* can only partially explain the TIL. Thus, it is worthwhile to investigate whether other non-drug metabolic influences exist or not.

Untargeted metabolomics is a flourishing unbiased approach for the systematic and global analysis of small molecule metabolites in biological systems (20–22). Lipidomic is a large branch of metabolomics aimed at studying how lipid metabolism is perturbed by biological stimuli and has been extensively applied to capture total lipids in the body (23, 24). Lipids are an important class of compounds widely found in living organisms and play a key role in a range of life activities. As early as 1979, studies have found that compared with normal mature neutrophils, the lipid composition of acute lymphoblastic leukemia cells was changed, with a decrease in total cholesterol and cholesterol–phospholipid ratio and an increase in the percentage of unsaturated fatty acids. Such lipid changes may be the same as normal immature myeloid cells (25). Several studies have shown extensive changes in the plasma (26) and bone marrow (27) lipid profiling of acute lymphoblastic leukemia patients. Recently, studies have also found that hypertriglyceridemia is a risk factor for early death in acute promyelocytic leukemia (28). Yu et al. found that UC patients often had an imbalance in lipid homeostasis, in which triglycerides and phosphatidylcholine were significantly reduced (29). Lipidomic has been universally used to understand the pathogenesis of IBD and diseases of the blood system (1, 30) and has shown potential for the classification of disease subtypes and assessment of therapeutic response (1, 31). However, no lipidomic studies on TIL in IBD patients have been reported. Therefore, we proposed this exploratory study to analyze the plasma lipidomic profile in Chinese IBD patients prescribed thiopurines after NUDT15 genetic screening. The identified biomarkers were validated in an independent validation cohort. We established a random forest (RF) model based on six lipids that could well distinguish leukopenia patients from non-leukopenia patients.

## 2. Material and methods

### 2.1. Chemicals and reagents

HPLC grade acetonitrile (ACN) and methanol (MeOH) were obtained from Fisher Scientific (Fair Lawn, NJ, USA), and 2-propanol (IPA, LC-MS grade) was purchased from Merck (Darmstadt, Germany). Methyl tert-butyl ether (MTBE, HPLC grade) and ammonium acetate (AmAc) were purchased from Sigma–Aldrich (St. Louis, MO. USA). Ultrapure water (18.2 MΩ cm at 25°C) was obtained from a Millipore Direct-^®^Q ultrapure water system (Billerica, MA, USA).

### 2.2. Patient recruitment and study design

This is a prospective observational trial. The study was conducted at the Sixth Affiliated Hospital of Sun Yat-sen University. This center has a large number of IBD patients from different regions of China. The study was approved by the Clinical Research Ethics Committee of the Sixth Affiliated Hospital of Sun Yat-sen University and was registered at http://www.chictr.org.cn/ (ChiCTR2100050295).

Patients were recruited between 1 July 2018 and 31 January 2022. Only those patients between the ages of 14 and 75 years with a definite diagnosis of IBD who opted for thiopurine therapy were included. The diagnosis of IBD was based on endoscopic, clinical, radiological, histopathological, and/or surgical findings according to current guidelines (7). Disease location and behavior were categorized in the light of the Montreal classification. The exclusion criteria included patients with *NUDT15 C415T* homozygotes (TT genotype), administration of methotrexate or cyclosporine; insufficient function of the heart, liver, or kidney; pregnancy; and blood transfusion and active infection. Dose reduction could be applied to patients who developed adverse events, for example, leukopenia [white blood cell count (WBC) <3.5 × 109/L], rash, hepatotoxicity, flu-like symptoms, gastric intolerance, pancreatitis, or others. If the laboratory abnormalities do not subside, the treatment will be discontinued.

*NUDT15 C415T* genotyping for each patient was conducted by Guangzhou KingMed Diagnostics Group Co., Ltd. For wild-type carrier (CC) patients, an initial dose of 1.0 mg/kg per day for AZA or 0.5 mg/kg per day for MP was gradually increased to a target dose of 2.0 mg/kg or 1.0 mg/kg, respectively. The dose was halved for those carrying the CT genotype. Usually, blood samples were taken on routine follow-up with the examination of complete blood cell count and Comprehensive Metabolic Panel, when patients were fasting. Sometimes the blood samples were taken for further consultation when patients were not required to be fasting. In general, 5 ml of venous blood samples (EDTA anticoagulation) were collected at the onset of leukopenia or at least 4 weeks after stable dosing.

### 2.3. Sample preparation

Approximately 300 μL of cold MeOH was added to 40 μL of plasma sample followed by the addition of 1 mL of MTBE and vortex mixing (10 s before and after adding MTBE). After vibrating the mixture for 15 min and the addition of 300 μL H_2_O, a two-phase system was formed. Subsequently, the mixture was equilibrated on ice for 10 min followed by centrifugation at 15,000 rpm under 4°C. Approximately 400 μL of supernatant was lyophilized and stored at −80°C for LC-MS/MS analysis. To ensure the repeatability of the assay, QC samples were prepared the same as the test sample for analyses as well.

### 2.4. UPLC-QExcative/MS data acquisition

A Thermo Scientific Dionex Ultimate 3000 UPLC-ESI-Q Exactive system was used for untargeted lipidomic in full MS/ddMS2 modes. After sample randomization, freeze-dried samples were reconstituted in ACN/IPA/H2O (65:30:5, v/v/v/) containing 5 mM AmAc, and 10 μL was injected in the Waters Acquity BEH C18 column (100 mm × 2.1 mm, 1.7 μm) coupled to an Acquity UPLC BEH C18 1.7 μm VanGuard pre-column (5 mm × 2.1 mm) for both positive ion mode and negative ion mode. Mobile phases A and B were ACN/H2O (60:40, v/v) and IPA/ACN (90:10, v/v), respectively, both containing 10 mM AmAc. The linear elution gradient started with 68% A and held for 1.5 min, then linearly reduced to 15% at 15.5 min, and then to 3% A at 15.6 min, and kept for 2.4 min. The gradient was returned to 68% A at 18.1 min and was maintained for 1.9 min to equilibrate the column. The flow rate was 0.26 mL/min. The column temperature was set at 55°C, and the temperature of the sampler was set at 8°C.

MS was performed with a heated ESI source in positive and negative modes, respectively. The spray voltage was set to 3.5 KV in the positive mode and 3.0 kV in the negative mode. Nitrogen was used as sheath gas and auxiliary gas and was set to 45 and 10 arbitrary units, respectively. The ion transfer capillary temperature was set at 300°C. Full-MS scan and data-dependent MS/MS (ddMS2) have resolutions of 70,000 and 17,500, respectively. The AGC target was 3 × 10^6^ ions capacity in the full-MS scan, and their value was 1 × 10^5^ ions capacity in ddMS2. The maximum IT was 100 ms in the Full-MS scan, and the value in ddMS2 was 50 ms. The TopN (N, the number of top most abundant ions for fragmentation) was 15. To capture as much informative MS/MS data as possible, three normalized collision energies (NCE) were set to 25, 35, and 45 eV in both positive and negative modes. MS data were acquired in the scan range of m/z 133.4–2,000.

### 2.5. Data processing and statistical analysis

Lipidomic data processing was done using lipid search software (Thermo Scientific, San Jose, CA, United States) according to our previous report (32). Then, a normalization approach (systematic error removal using random forest, SERRF) based on using quality control pool samples was performed (33). R package muma (34) was used for univariate and multivariate statistical analysis, and Shapiro–Wilk's test for normality was performed for each variable (lipid species). If the variable fits a normally distributed, then Welch's *t*-test was performed; otherwise, Wilcoxon–Mann–Whitney *U*-test was performed (35). The fold change (FC) of each variable between the two groups was also calculated. A *p*-value of < 0.05 was considered to be statistically significant.

### 2.6. Differential lipid species screening and pathway analysis

Principal component analysis (PCA) and orthogonal partial least squares-discriminant analysis (OPLS-DA) were performed using SIMCA-P software (version 13.0; Umetrics, Kinnelon, NJ, United States). PCA was used to identify the data in an unsupervised state to investigate the clustering within each group. OPLS-DA was used to analyze the data under supervised conditions to observe the separation of data between groups. Variable importance in projection (VIP) values was employed to filter variables from the OPLS-DA model. Molecular features with VIP > 1.0, fold change of >1.2 or <0.8333, and a *p*-value of < 0.05 were considered to be potential differential lipid species. Network analysis with potential differential lipid subclass and visualization of metabolic pathways was achieved by Metscape running on Cytoscape 3.7.2.

### 2.7. Model construction for biomarker discovery

Least Absolute Shrinkage and Selection Operator (LASSO)-based variable selection was performed using the Python package “sklearn” and R package “glmnet.” “Sklearn” was used for 50 times LASSO, counts the frequency of non-zero variables in 50 LASSO, and sorts them from high to low. Variables with frequencies not <30 were selected for RF classification analysis by using the R package “randomForest.” We conducted 5-fold cross-validation to get an unbiased estimate of the model performance. We randomly divided the dataset into five parts. In each fold of cross-validation, we used one part as the test set and the remaining four parts as the training set. We used this model for testing on the test set to calculate the classification accuracy. In addition, we validated our model with an independent external validation cohort as a validation set. LASSO-logistic regression analysis was conducted using the R package “glmnet.” SVM analysis was performed using the R package “e1071”.

## 3. Results

### 3.1. Demographics and serum biochemical analysis

Demographics and basic clinical information are shown in Table 1. In both cohorts, female patients were more likely to develop leucopenia compared with male patients [discovery cohort, *P* = 0.004, odds ratio (OR) 1.89, 95% confidence interval (CI) 0.59~6.02; validation cohort, *P* = 0.018, OR 2.78, 95% CI 0.72~10.53]. There was a significant association of *NUDT15 C415T* genotypes with leucopenia (discovery cohort, *P* = 0.003; validation cohort, *P* = 0.048). No statistical differences were found in terms of age, disease type, and drug combination between IBD patients with and without thiopurine-induced leucopenia from the discovery cohort and the validation cohort.

**Table 1:** Clinical demographics of the subjects.

	**Discovery cohort (*****N** =* **127)**			**Validation cohort (*****N** =* **100)**		
**Racteristics**	**leukopenia** ***N** =* **27**	**Non-leukopenia** ***N** =* **100**	***p***	**leukopenia** ***N** =* **26**	**Non-leukopenia** ***N** =* **74**	***p***
Gender, Male, *n* (%)	15 (55.6)	82 (82)	0.004	15 (57.7)	60 (81.1)	0.018
**Age**, ***n*** **(%)**			0.30			0.37
< 18	1 (3.7)	3 (3)		1 (3.8)	1 (1.4)	
18~40	22 (81.5)	88 (88)		19 (73.1)	58 (78.4)	
>40	4 (14.8)	9 (9)		6 (23.1)	15 (20.2)	
**Diagnosis**, ***n*** **(%)**						
CD	26 (96.3)	99 (99)	0.32	26 (100)	73 (98.6)	0.55
UC	1 (3.7)	1 (1)		0 (0)	1 (1.4)	
**NUDT15 rs116855232**, ***n*** **(%)**						
CC	19 (70.4)	92 (92)	0.003	19 (73.1)	66 (89.2)	0.048
CT	8 (29.6)	8 (8)		7 (26.9)	8 (10.8)	
**Medication**						
AZA	21 (77.8)	91 (91)	0.06	23 (88.5)	71 (95.9)	0.17
6-MP	6 (22.2)	9 (9)		3 (11.5)	3 (4.1)	
Thiopurines dose, AZA mg/kg/day, median (IQR)	1.2 (0.9–1.7)	1.6 (1.1–1.9)		1.5 (1.1–2.0)	1.6 (1.11.9)	
**Co-medication**			0.052			0.27
Steroids, *n* (%)	1 (3.7)	13 (13)		2 (7.7)	6 (8.1)	
Infliximab, *n* (%)	3 (11.1)	7 (7)		6 (23.1)	0 (0)	
5-aminosalicylic acid, *n* (%)	1 (3.7)	0 (0)		0 (0)	2 (2.7)	
EEN, *n* (%)	3 (11.1)	3 (3)		0 (0)	0	

AZA, azathioprine; CD, Crohn's disease; IBD, inflammatory bowel disease; IQR, interquartile range; OR, odds ratio; UC, ulcerative colitis; EEN, Exclusive Enteral Nutrition.

### 3.2. Lipid profiling and differential lipid species analysis between CD patients with and without thiopurine-induced leucopenia

**Figure 1:**
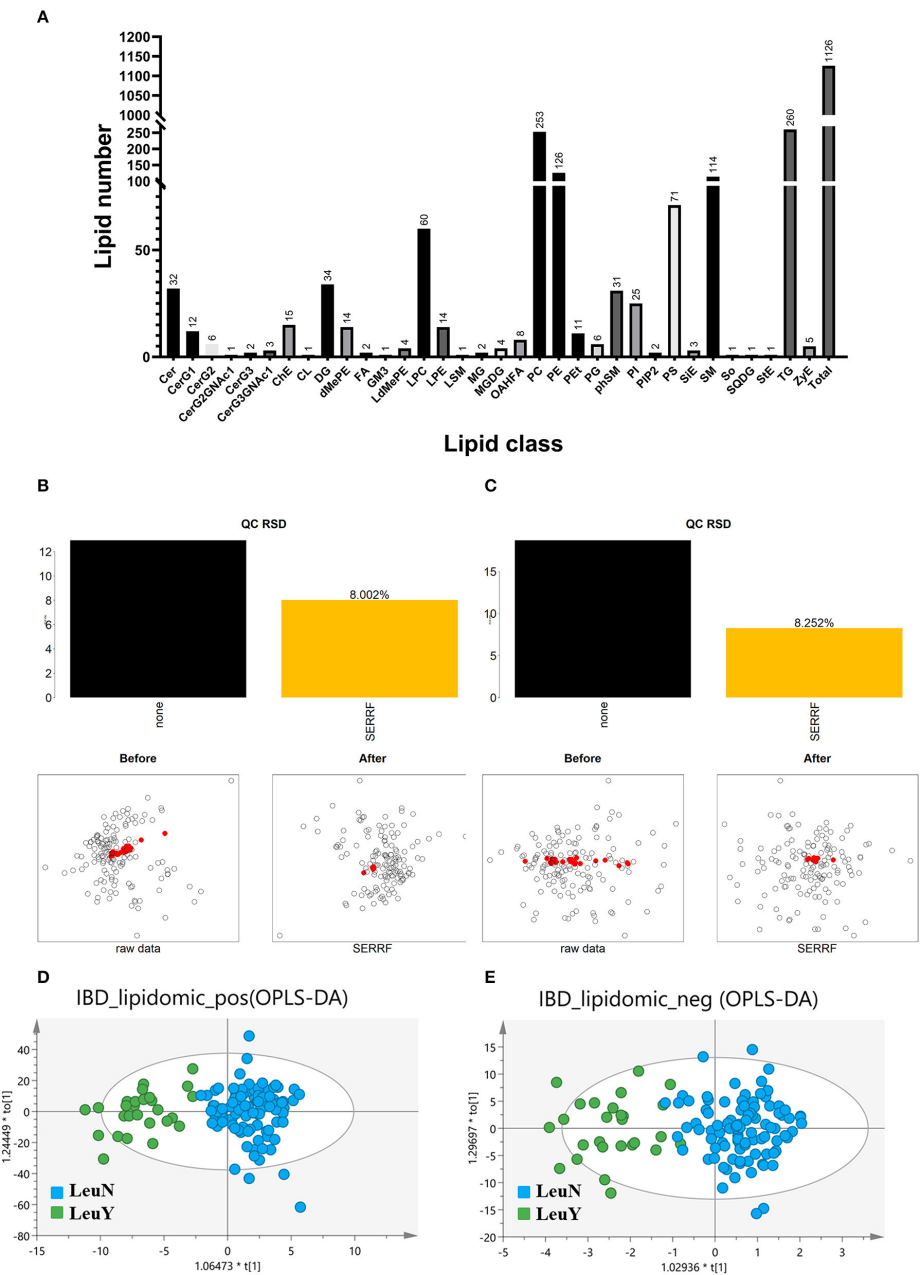
Untargeted lipidomic analysis. **(A)** Number of detected lipids based on a untargeted lipidomic. SERRF normalization to remove the unwanted systematic variations for positive **(B)** and negative **(C)** mode. The red dots represent QC samples, which are clustered together after SERRF normalization, indicating that systematic errors are eliminated. Scores scatter plot of OPLS-DA in both positive **(D)** and negative **(E)** mode. LeuN, the non-leukopenia group. LeuY leukopenia group.

The typical chromatograms in the positive and negative ion modes are shown in Supplementary Figure S1. In untargeted lipidomic analysis, we examined plasma samples by positive and negative ion modes with two injections and detected 1,126 lipid species including 952 in the positive ion mode and 174 in the negative ion mode from 127 patients in the discovery cohort (Figure 1A).

The raw data were normalized using SERRF normalization to remove the unwanted systematic variations. The results showed that SERRF reduced the average technical errors for the positive and negative ion modes to 8.002 and 8.252% relative standard deviation (RSD), respectively (Figures 1B, C). The aggregation of QC samples was improved, and the RSD of QC was reduced after normalization by SERRF, indicating that SERRF effectively reduced the systematic variation of primary effect data.

An OPLS-DA model was developed to provide a lipid analysis profile between the leukopenia and non-leukopenia groups in positive and negative ion modes, respectively (Figures 1D, E). The key parameter R2 was applied to evaluate the discriminative power of the model. R2 is 0.779 in the positive ion mode and 0.750 in the negative ion mode. It is indicated that the OPLS-DA model established in positive and negative ion modes can distinguish the two groups well. In addition, the results of the 200-item permutation test proved that the fitted OPLS-DA model was not overfitted. Molecular features with VIP > 1.0, fold change >1.2 or <0.8333, and *p* < 0.05 were used as the screening criteria for potential differential lipid species. A total of 119 plasma lipid species were considered to be the main cause of differences between the leukopenia and the non-leukopenia group.

### 3.3. Perturbed metabolic pathway

**Figure 2:**
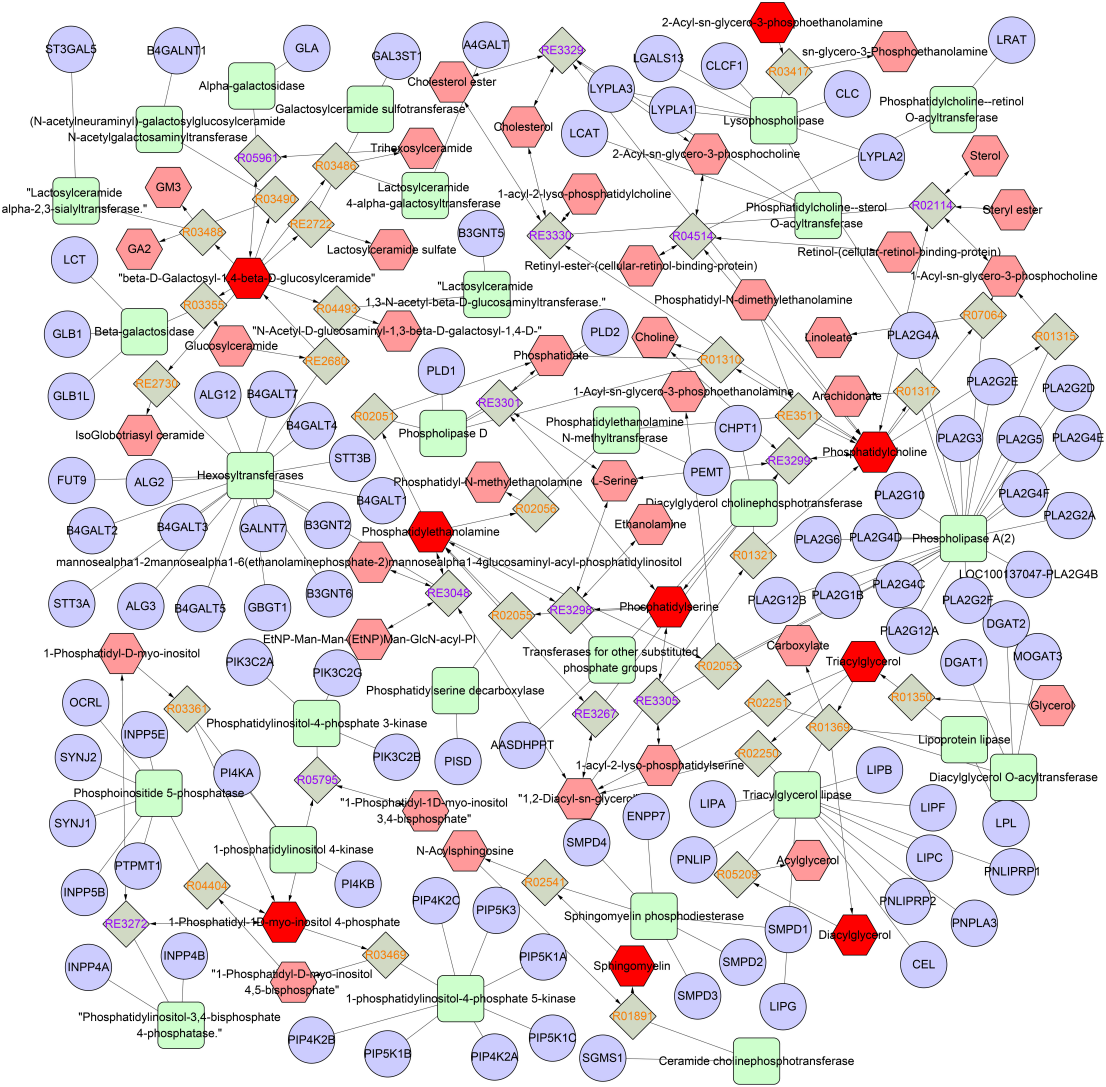
Network of the remarkably perturbed metabolic pathways by MetScape analysis. The red hexagons indicate the differential lipid metabolites identified in our study. Moreover, the pink ones are the involved metabolites that have not been identified in our study. The diamond ones are the involved reaction. The ellipse ones are the involved gene, and the round rectangle ones are the enzyme.

To map the lipid species pathway of identified 119 lipid species from TIL in IBD patient study, network analysis was conducted and metabolic pathways were visualized, showing that five metabolic pathways were involved, including glycosphingolipid metabolism, glycosphingolipid biosynthesis–globoseries, glycosphingolipid biosynthesis–ganglioseries, Ggycerophospholipid metabolism, and arachidonic acid metabolism (Figure 2).

### 3.4. Independent validation of specific lipidomic signatures

**Figure 3:**
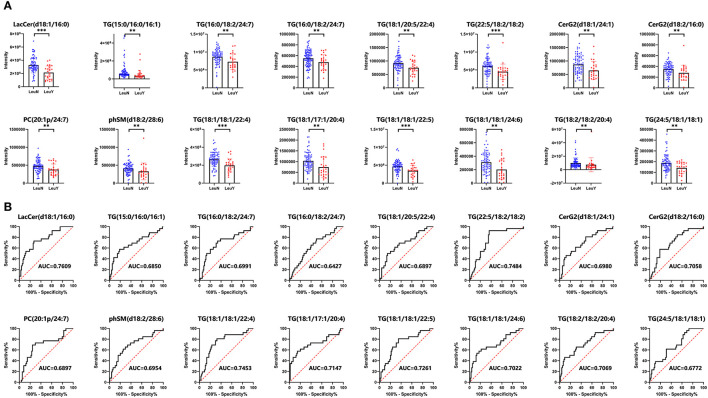
A total of 16 lipid species were further validated by a prospective, observational study. **(A)** Dot and bar plots of levels of 16 lipid species including LacCer (d18:1/16:0), TG (22:5/18:2/18:2), TG (18:1/20:5/22:4), TG (18:1/20:4/22:4), TG (15:0/16:0/16:1), TG (16:0/18:2/24:7), CerG2 (d18:1/24:1), TG (18:1/18:1/22:4), TG (18:1/17:1/20:4), TG (24:5/18:1/18:1), TG (18:1/18:1/24:6), TG (18:1/18:1/22:5), TG (18:2/18:2/20:4), phSM (d18:2/28:6), PC (20:1p/24:7), and CerG2 (d18:2/16:0). **(B)** ROC curves of the 16 lipid biomarkers in leukopenia IBD and non-leukopenia IBD patients. The AUC of each biomarker is indicated. LeuN, the non-leukopenia group. LeuY, leukopenia group. ^**^p < 0.01, ^***^p < 0.001 by the *t-*test or Wilcoxon–Mann–Whitney *U*-test.

We further identified lipidomic alterations in an independent validation cohort of 100 subjects, which included 74 non-leukopenia patients and 26 leukopenia patients. The findings validated that 16 of the 119 metabolites had an FDR < 0.05. These 16 lipid species, including LacCer (d18:1/16:0), TG (22:5/18:2/18:2), TG (18:1/20:5/22:4), TG (18:1/20:4/22:4), TG (15:0/16:0/16:1), TG (16:0/18:2/24:7), CerG2 (d18:1/24:1), TG (18:1/18:1/22:4), TG (18:1/17:1/20:4), TG (24:5/18:1/18:1), TG (18:1/18:1/24:6), TG (18:1/18:1/22:5), TG (18:2/18:2/20:4), phSM (d18:2/28:6), PC (20:1p/24:7), and CerG2 (d18:2/16:0), have high robustness and reproducibility and can be considered as potential biomarkers for IBD. Figure 3A demonstrates the different levels of the 16 lipid species in plasma in the leukopenia and non-leukopenia group. The ROC curves of the 16 lipid species are shown in Figure 3B.

### 3.5. Well distinguish the leukopenia group from the non-leukopenia group by the six lipid species established models

**Figure 4:**
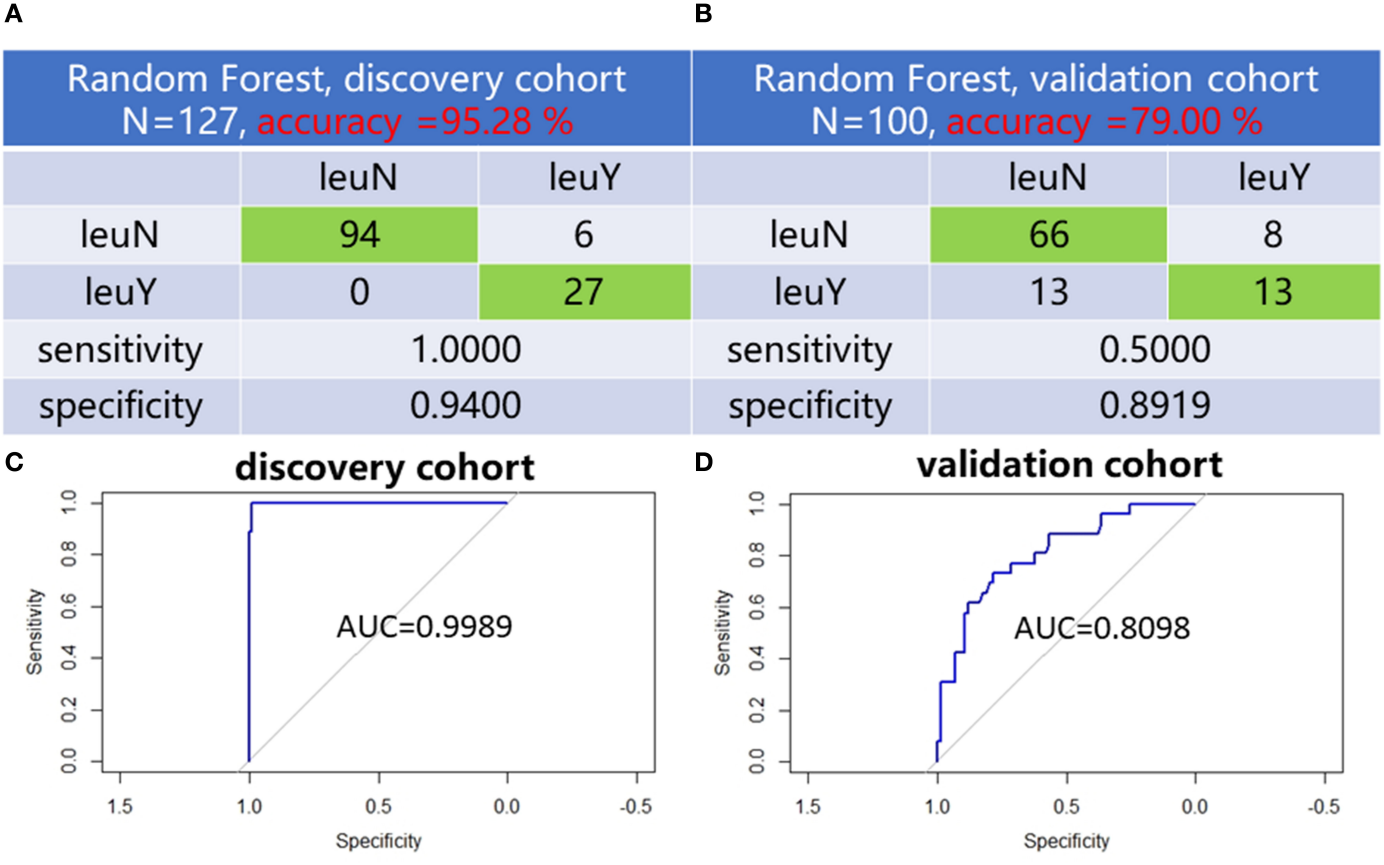
Six lipid species established distinguish models. Confusion matrix of random forest evaluation on the model from discovery cohort **(A)** and validation cohort **(B)**. Analysis of RF model-based ROC curves of six lipid species in leukopenia patients and non-leukopenia patients from discovery cohort **(C)** and validation cohort **(D)**. leuN, non-leukopenia group. leuY, leukopenia group.

LASSO was used to further narrow the number of differential lipid species to suit actual clinical needs. To remove the bias caused by randomly dividing the dataset, we performed 50 times LASSO-based variable selections. An RF regression model was fit by using six lipid species with a frequency more than 30 times in the 50 times LASSO-based variable selections to distinguish the leukopenia group from the non-leukopenia group (Table 2). Six lipid species including LacCer (d18:1/16:0), TG (15:0/16:0/16:1), TG (16:0/18:2/24:7), TG (18:1/20:4/22:4), TG (18:1/20:5/22:4), and TG (22:5/18:2/18:2) can clearly distinguish the two groups. In the discovery cohort, the model had a classification accuracy of 95.28%, and the 95% confidence interval (CI) is (0.9000, 0.9825). The confusion matrix of RF for the discovery cohort and validation cohort is shown in Figures 4A, B. The ROC curve showed that AUC values were equal at 0.9989 (Figure 4C). In the validation cohort, the model had a classification accuracy of 79.00%, and the 95% CI is (0.6971, 0.8651). The ROC curve showed that AUC values were equal at 0.8098 (Figure 4D). To further test the classification power of the six lipid species, we also established two classification algorithms, logistic and support vector machine classification, and obtained similar results to the random forest (Supplementary Figure S2).

**Table 2:** Lipid metabolites distinguish IBD patients who developed leukopenia from those who do not.

**Lipid species**	**Formula**	**RT [min]**	**Frequency**	***P***	**FDR**	**FC**
LacCer (d18:1/16:0)	C46H88O13N1	11.42858	50	7.98E-05	0.0083	1.53
TG (15:0/16:0/16:1)	C50H98O6N1	17.49223	45	0.0051	0.038	1.22
TG (16:0/18:2/24:7)	C61H101O6	17.47783	43	0.0026	0.030	1.19
TG (18:1/20:4/22:4)	C63H105O6	17.81924	49	0.0036	0.033	1.20
TG (18:1/20:5/22:4)	C63H103O6	17.50232	50	0.0039	0.033	1.24
TG (22:5/18:2/18:2)	C61H104O6N1	16.97768	50	1.72E-04	0.0084	1.35

### 3.6. Blood biochemical tests for lipoprotein, cholesterol, and triglyceride

**Figure 5:**
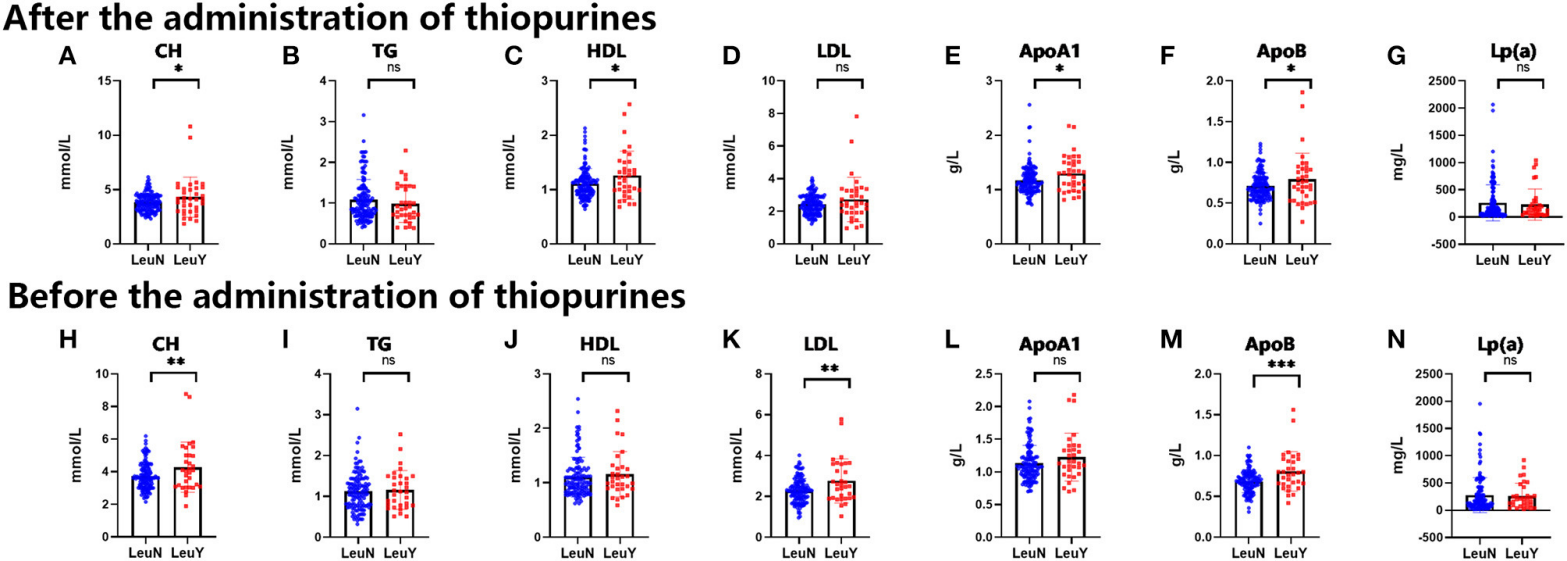
Blood biochemical tests of lipoprotein, cholesterol and triglyceride before and after administration of thiopurine in IBD patients. The level of CH, TG, HDL, LDL, ApoA1, ApoB, Lp(a) after administration of thiopurine in IBD patients **(A–G)**. The level of CH, TG, HDL, LDL, ApoA1, ApoB, Lp(a) before administration of thiopurine in IBD patients **(H–N)**. LeuN, non-leukopenia group. LeuY, leukopenia group. ^*^p < 0.05, ^**^p < 0.01, ^***^p < 0.001 by the t-test.

We performed the *t-*test on total cholesterol (CH), triglycerides (TG), high-density lipoproteins (HDL), low-density lipoproteins (LDL), Apolipoprotein A1 (ApoA1), Apolipoprotein B (ApoB), and lipoprotein α(Lp(a)) levels. After the administration of thiopurines, CH (*p* = 0.02), HDL (*p* = 0.01), ApoA1 (*p* = 0.01), and ApoB (*p* = 0.03) had higher levels in the leukopenia group compared to the non-leukopenia group. TG, LDL, and Lp(a) were no different between the two groups (Figures 5A–G). Before thiopurines treatment, CH (*p* = 0.007), LDL (*p* = 0.001), and ApoB (*p* = 0.0003) had higher levels in the leukopenia group compared to the non-leukopenia group, and there were no differences in the others between the two groups (Figures 5H–N), that is there was no difference in blood total TG levels between two groups, either before or after the treatment.

## 4. Discussion

IBD is a chronic inflammatory gastrointestinal disease, and MP/AZA has been widely used in IBD patients (8). However, serious adverse events, leukopenia, limit its clinical application. TPMT and NUDT15 are thought to be pivotal enzymes in thiopurine metabolism and are relevant to TIL (13). Leukopenia still occurs in about 20% of patients, even after accounting for *TPMT* and *NUDT15* (16). The plasma lipid profiles of IBD patients with thiopurine treatment have not been systematically investigated. In this study, we for the first time characterized a detailed plasma lipid profile of TIL after NUDT15 C415T screening in Chinese IBD patients.

We performed untargeted lipidomic analysis to explore lipid level changes *in vivo* during TIL in two independent cohorts and finally screened out six lipid species using machine learning-related algorithms that could well distinguish patients from the leukopenia group and the normal group. These six lipid species performed excellently in the three algorithms of lasso-logistic, support vector machine, and random forest, proving the screening performance of these six lipids. In addition, it is worth noting that the random forest algorithm has the best discrimination effect with the established model, with an accuracy of 95.28% (discovery cohort) and 79.00% (validation cohort), and ROC showed that AUC value is equal at 0.9989 (discovery cohort) and 0.8098 (validation cohort).

Lactosylceramides (LacCer) are the most important and abundant type of diosylceramides and belong to the class of glycosphingolipids (36). Approximately 70% of the glycosphingolipids in human neutrophils are lactosylceramide (37, 38). The number of glycolipids changes significantly during leukocyte differentiation, indicating that these molecules are involved in biological functions (37). The previous study found that the lactosylceramide-enriched glycosphingolipid signaling domain mediates superoxide production in human neutrophils (39). Therefore, TIL may be related to the abnormal expression of LacCer on neutrophils. Further research is needed to confirm.

Five of the six lipids used for modeling belong to triglycerides, indicating that the leukopenia in IBD patients caused by thiopurine may be related to the metabolism of triglycerides. A study of the relationship between the abnormal lipid profile and inflammation and progression of myelodysplastic syndrome to acute leukemia (patients = 11,071) found that elevated triglycerides were dramatically related to the diagnosis of acute leukemia in patients with myelodysplastic syndrome (40). A retrospective study containing 1,412 cases found that patients with acute promyelocytic leukemia (APL) had higher triglyceride levels than non-APL and control subjects. There was a positive correlation between triglyceride levels and WBC, which is consistent with the findings of this study (28). Further study found that the interaction between hypertriglyceridemia and acute promyelocytic leukemia is mediated by the cooperation of peroxisome proliferator-activated receptor-alpha with PML/RAR alpha fusion protein on the super enhancer (28). However, we observed that there was no difference in blood total TG levels between the two groups, either before or after the treatment. This difference implies that the levels of individual specific TG molecules rather than total TG levels are involved in the TIL process, indicating their potential importance. In addition, our results showed that patients with leukopenia had higher blood CH and ApoB levels than normal patients before and after treatment. A study found that the lipid-lowering drug gemfibrozil induces leukopenia via PPAR-α in mice (41). Further investigation is necessary to clarify whether thiopurine-inducedleukopenia is also mediated through PPAR-α.

Our current study has several limitations. We only investigated the lipid profile of patients with IBD when leukopenia occurred, and there are limitations to single-point measurement. Although we additionally collected an independent validation cohort to ensure that our results are stable and reliable, repeating-measures across several visits on the same patient are a good way to understand the intra-subject variation at the time of diagnosis and its evolution process. In addition, the six lipid species were not validated from populations of other parts of Asia.

## 5. Conclusion

In conclusion, our results revealed the essential lipid species and pathways that could contribute to distinguishing the TIL in the IBD after NUDT15 C415T screening. Six lipid species including LacCer (d18:1/16:0), TG (15:0/16:0/16:1), TG (16:0/18:2/24:7), TG (18:1/20:4/22:4), TG (18:1/20:5/22:4), and TG (22:5/18:2/18:2) were screened and can clearly distinguish the two groups. The results showed a tight link between plasma lipid profiles and TIL, which was most pronounced in LacCer and TG. With powerful machine learning-based algorithms, including LASSO logistic regression, random forests, and support vector machine models, we were able to screen potential biomarkers for potential clinical applications. Our novel findings have enabled us to better understand the mechanisms of TIL and to develop models with clinical applications and new therapeutic strategies for the individualized treatment of thiopurines.

## Data availability statement

The raw data supporting the conclusions of this article will be made available by the authors, without undue reservation.

## Ethics statement

The studies involving human participants were reviewed and approved by the Clinical Research Ethics Committee of the Sixth Affiliated Hospital of the Sun Yat-sen University. Written informed consent to participate in this study was provided by the participants' legal guardian/next of kin.

## Author contributions

PL: conceptualization, data analysis, methodology, investigation, writing—original draft, and writing—review and editing. KC: conceptualization, methodology, writing—original draft, and writing—review and editing. TY, JM, and XZ: patient enrollment and follow up and writing—review and editing. ZH: resources, software, and visualization. LQ: collecting data. PH: supervision and resources. MH: supervision, funding acquisition, and writing—review and editing. XG: project administration, resources, and writing—review and editing.
