# Supplementary material for: Plasma lipidomic profiling of thiopurine-induced leukopenia after NUDT15 genotype-guided dosing in Chinese IBD patients
Source: Front Nutr. 2023 Jun 27;10:1138506. doi: 10.3389/fnut.2023.1138506 (PMC10333543; doi:10.3389/fnut.2023.1138506)
Supplement: Supplementary file 1 [file Data_Sheet_1.docx]

## Plasma lipidomic profiling of thiopurine-induced leukopenia after NUDT15 genotype-guided dosing in Chinese IBD patients

Pan Li^1,&^, Kang Chao^2,3,&^, Zhanhua Hu^1^, Lulu Qin^4^, Zhong Zuo^5^, Wen Xie^6^，Ting Yang^1^, Jing Mao^1^, Xia Zhu^1,2,3^, Pinjin Hu^2,3^, Xueding Wang^1^, Xiang Gao^2,3,*^, Min Huang^1,*^

^1^ Guangdong Provincial Key Laboratory of New Drug Design and Evaluation, School of Pharmaceutical Sciences, Sun Yat-sen University, Guangzhou, China

^2^ Department of Gastroenterology, The Sixth Affiliated Hospital, Sun Yat-sen University, Guangzhou, China

^3^ Guangdong Institute of Gastroenterology, Guangdong Provincial Key Laboratory of Colorectal and Pelvic Floor Diseases, Supported by National Key Clinical Discipline, Guangzhou, China

^4^ School of Pharmaceutical Sciences, Guangdong Pharmaceutical University, Guangzhou, China

^5^ School of Pharmacy, Faculty of Medicine, The Chinese University of Hong Kong, Hong Kong SAR, China

^6^ Department of Pharmacology and Chemical Biology, University of Pittsburgh, Pittsburgh, PA 15261, USA.


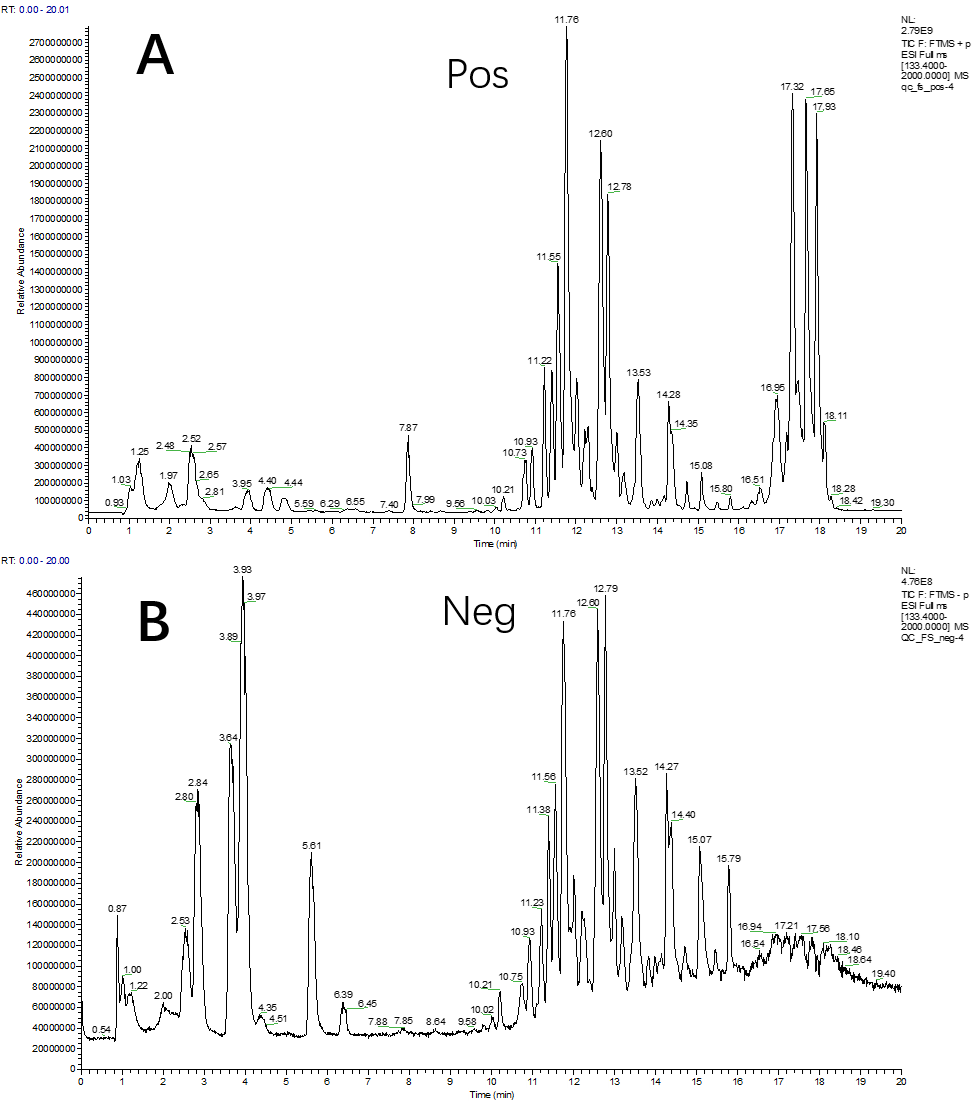


Figure S1. (A) Base peak intensity (BPI) chromatograms of plasma from a QC sample in positive ion mode. (B) Base peak intensity (BPI) chromatograms of plasma from a QC sample in negative ion mode.


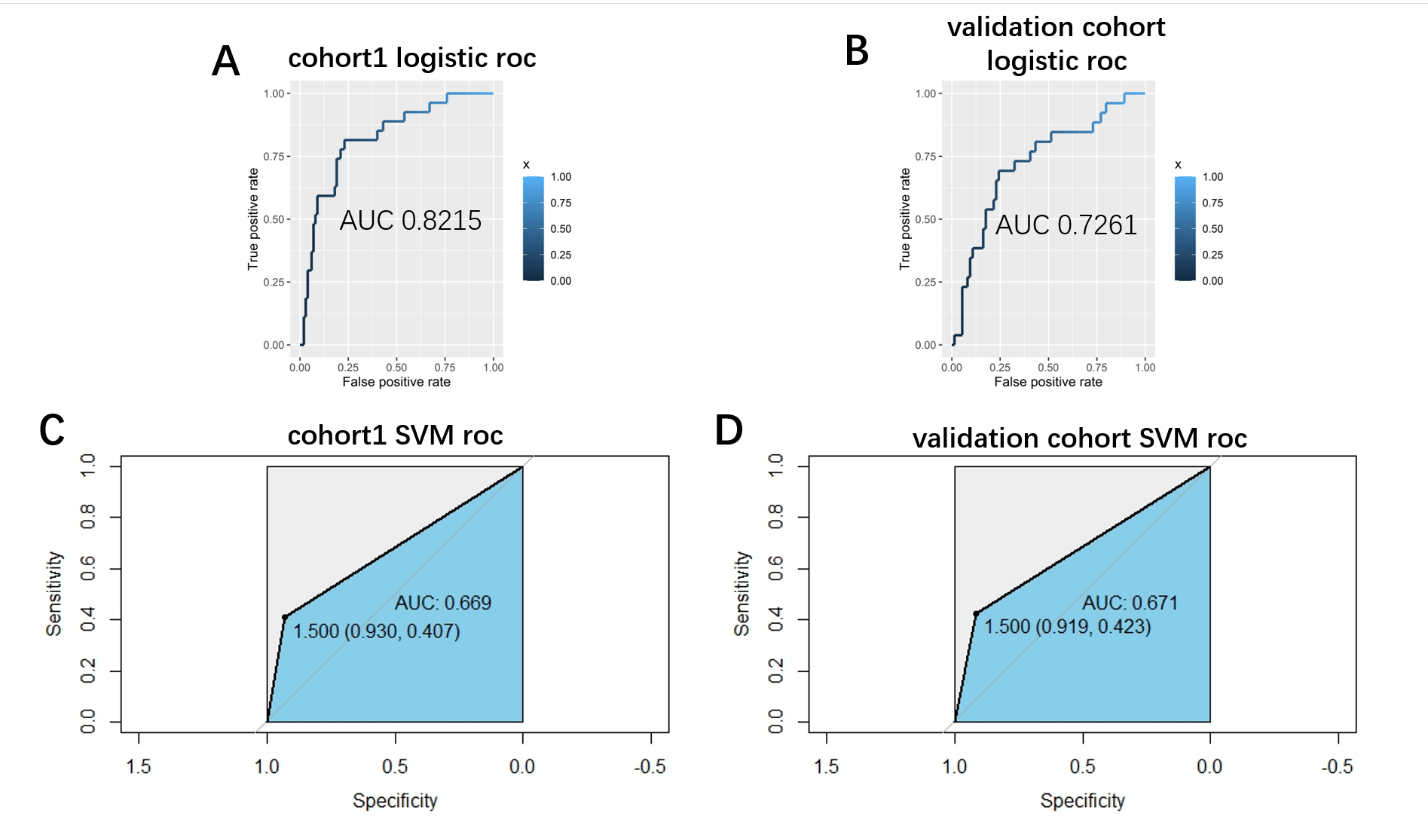


Figure S2 Logistic and support vector machine classification. Analysis of logistic model based ROC curves of six lipid species in leukopenia patients and non-leukopenia patients from discovery cohort(A) and validation cohort(B). Analysis of SVM model based ROC curves from discovery cohort(C) and validation cohort(D).
